# From visual attention to behavioural intention: A relational framework of culturally creative desserts in museum communication

**DOI:** 10.1371/journal.pone.0347418

**Published:** 2026-04-13

**Authors:** Zichen Ke, Xiang Meng, Jingzong Xu, Muhizam Mustafa

**Affiliations:** 1 School of the Arts, Universiti Sains Malaysia, Gelugor, Penang, Malaysia; 2 School of the Art and Design, Anyang Institute of Technology, Anyang, Henan, China; Sichuan Agricultural University, CHINA

## Abstract

This study examines how the visual presentation of culturally creative desserts (CCDs), modelled after Chinese museum artefacts, relates to potential visitors’ visit-related behavioural intention under first-exposure, image-mediated conditions. Drawing on the AIDA model, this study develops a four-stage framework (VCVB: perceived Visual Attention, Curiosity, Visit Motivation, Behavioural Intention) to examine whether visual attention is directly associated with visit motivation in visually mediated museum contexts. A survey using image-based stimuli was conducted with 205 respondents and analysed using Partial Least Squares Structural Equation Modelling (PLS-SEM). The results reveal an indirect-only pattern: perceived visual attention is positively related to curiosity, curiosity to visit motivation, and visit motivation to behavioural intention, whereas the direct association between visual attention and visit motivation is not significant. These findings indicate that the commonly assumed direct association between visual attention and motivational orientation does not hold under first-exposure, image-mediated conditions. Instead, visual attention operates indirectly through epistemic curiosity and visit motivation, suggesting that visually driven attention alone is insufficient to generate visit-related intention.

## 1. Introduction

**Fig 1:**
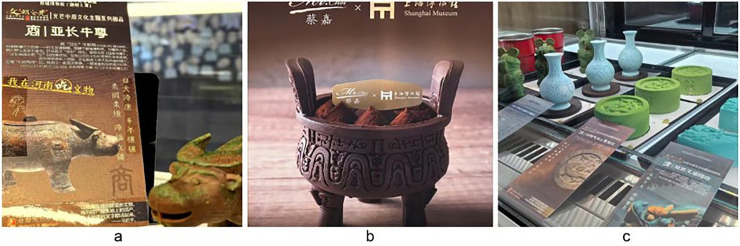
CCDs inspired by museum artefacts (Photographs by the author: (a) Yinxu Museum, (b) Shanghai Museum, and (c) Henan Museum).

A notable visual trend has recently emerged in Chinese public museums: desserts that replicate artefacts with high fidelity and are often encountered on social media before the originals are seen in person (see Fig 1). Widely circulated through social media, these culturally creative desserts (CCDs) have become a new form of visually driven cultural communication, where “eating with the eyes” precedes any actual tasting [1,2]. Despite their growing popularity as visual communication tools, the stage-based relationships linking CCD imagery, perceived visual attention, and subsequent museum-related intention formation remain insufficiently specified, particularly in terms of how these relationships are structured rather than causally established or behaviourally validated. This phenomenon has not emerged in isolation, but is closely intertwined with institutional transformations in China’s public museum sector, in which policy-driven audience expansion has contributed to changes in communicative strategies and forms of public communication through which visual content is presented to the public [3–5].

The emergence of CCDs is closely linked to broader institutional transformations in China’s public museum sector. Since the nationwide free-admission policy in 2008, museums have been required to reach wider audiences while operating under non-profit mandates. Free admission expanded public access to museums, while subsequent policy guidelines explicitly encouraged the development of cultural and creative products and cross-sector collaborations [6,7]. Together, these policies have fostered an institutional environment in which museums increasingly repackage cultural content into visually engaging, public-facing formats, extending beyond their traditional exhibition-oriented roles [3,8,9]. Within this broader shift, CCDs have emerged as a prominent manifestation of museums’ increasingly visual communication strategies, through which cultural content is re-presented to reach wider publics.

CCDs refer to desserts whose forms, colours, and surface details closely replicate iconic artefacts, such as bronze vessels or jade seals, and which are officially developed or endorsed by non-profit museums. As edible cultural products, CCDs combine food form with artefact-inspired visual design. Their visually striking appearance makes these food items highly shareable on social media, enabling audiences to encounter and respond to them even before visiting the museum.

Existing research offers two complementary yet incomplete perspectives. On the one hand, studies on food-related experiences have largely been dominated by a post-consumption, multisensory paradigm, explaining behavioural responses primarily through taste-based enjoyment [10–13]. Such a framework is less suited to CCDs, which are typically encountered visually prior to any physical consumption. On the other hand, research on image-based communication demonstrates that visual salience and novelty effectively capture attention, yet these studies often abstract visual cues from the specific context of museum-endorsed, edible cultural artefacts [14–18]. As a result, a critical gap exists at the intersection of these literatures: the stage-based relationships through which visually encountered, museum-endorsed edible artefacts are associated with cultural participation remain insufficiently articulated. For the purposes of this study, cultural participation is operationalised at the level of visit-related behavioural intention (BI), primarily referring to intention to visit the museum, while also including related responses such as sharing and recommendation.

To address this gap, this study adapts the classic AIDA model (Attention, Interest, Desire, Action) to a non-profit, visually driven museum context. In this setting, interest is more precisely conceptualised as epistemic curiosity, and desire as visit motivation. This reconceptualisation results in a four-stage framework comprising perceived Visual Attention (VA), Curiosity (CU), Visit Motivation (VM), and Behavioural Intention (BI), which is used to examine the stage-based relationships between perceived visual attention and visit-related behavioural intention under first-exposure conditions. This study focuses on how perceived visual attention is related to visit-related motivation within visually mediated, pre-consumption contexts.

Accordingly, this study addresses the following research questions: (1) How does perceived visual attention (VA) relate to curiosity (CU) and subsequently contribute to visit motivation (VM) and behavioural intention (BI) within the proposed VCVB framework under first-exposure conditions? (2) Does curiosity (CU) represent an indirect association between perceived visual attention and visit motivation (VM), and does visit motivation (VM) represent an indirect association between curiosity (CU) and behavioural intention (BI) within the proposed VCVB framework? To empirically examine the proposed VCVB framework, this study employs image-based survey stimuli and Partial Least Squares Structural Equation Modelling (PLS-SEM). Based on this analytical focus, this study contributes by showing that the commonly assumed direct association between visual attention and visit motivation does not hold in visually mediated museum contexts. Instead, visual attention is associated with visit motivation indirectly through epistemic curiosity. This suggests that, in visually mediated museum communication contexts, the relationship between attention and motivational orientation is not directly observed, but is structured through an epistemic component, thereby providing a more context-specific understanding.

## 2. Theoretical framework and research hypotheses

### 2.1. The role of AIDA in this study

Visually driven cultural experiences such as CCDs circulated online often elicit a more specific form of information-gap curiosity [19,20]. This curiosity is frequently associated with the visual and symbolic incongruity inherent in CCDs, in which an edible, everyday form visually references culturally significant artefacts [21]. Although substantial research has accumulated in the fields of visual communication, food-related cultural consumption, and museum studies, these insights remain largely fragmented across disciplinary boundaries. As a result, the stage-based relationships between visually encountered edible cultural products and museum-related intention formation under first-exposure conditions have not yet been systematically articulated in visually mediated museum contexts. Especially in digitally mediated environments, cultural content is often perceived visually prior to any embodied or sensory experience, and CCDs represent a typical example of such visually mediated hybrid cultural products.

In visual communication research, extensive attention has been devoted to examining how visual salience, novelty, aesthetic fluency, and multimodal processing influence audience attention allocation and immediate cognitive responses [17,22]. This body of work effectively explains how images capture attention and initiate early-stage perceptual processing [23,24]. However, it offers limited insight into how visual stimuli or perceptual responses are related to intention formation in relation to physically distant cultural institutions, leaving the relationship between perceptual response and institution-related intention formation largely under-theorised.

In food tourism research, scholars have extensively examined cultural meaning, authenticity, and identity construction, highlighting the symbolic functions of food as a cultural carrier and its role in enabling symbolic consumption and imagined authenticity [25–28]. This line of research has predominantly focused on sensory and affective responses following actual consumption or experiential engagement, emphasising taste-based enjoyment, emotional memory, and the formation of cultural identity [29,30]. By contrast, research remains limited on food products that are encountered solely through visual perception, prior to consumption, and that function primarily as symbolic references to external cultural objects rather than as objects of consumption themselves.

Museum studies have long emphasised object-based learning, materiality, and embodied experience, and widely recognise curiosity and object engagement as central drivers of museum visitation and learning [31–33]. However, this research tradition has largely focused on individuals who are already positioned as museum visitors, paying comparatively less attention to how visit motivation is generated prior to entry, particularly through digitally mediated, extra-institutional visual encounters involving non-traditional objects such as food. As a result, the stage-based relationships linking external, visually mediated stimuli with museum-oriented motivation remain insufficiently specified.

Therefore, in museum-related contexts, this incongruity can be interpreted as being associated with epistemic, object-oriented curiosity directed toward understanding the cultural meaning and origin of the referenced artefact [19,34]. This interpretation is consistent with contemporary developments in curiosity research, which emphasise the association between epistemic curiosity and clearly defined information gaps, the accompanying role of metacognitive feelings such as uncertainty, and expectations of potential informational gain. These processes are especially salient in visually mediated, digital-first encounter conditions [35–37]. In such contexts, when perceiving an edible object, viewers may interpret it as a reference to a museum artefact and further extend their meaning-making inference toward the cultural institution to which that artefact belongs. In this study, curiosity is not treated as a direct substitute for the broad AIDA category of “interest”, but is conceptualised as a more precisely bounded information-gap construct. This conceptualisation allows the relationship between visual salience and visit-oriented motivation to be analytically specified through an epistemic component within the proposed framework.

This study examines how, under first-exposure conditions, visually mediated perception is related to epistemic curiosity, museum-oriented visit motivation, and behavioural intention. Visual communication research explains how visual salience captures attention; food tourism research addresses symbolic meaning and imagined authenticity primarily in post-consumption contexts; and museum studies emphasise object-based curiosity while often presupposing prior visitor positioning. Therefore, the present study uses AIDA as a broad heuristic scaffold, while shifting the analytical focus from stage labels themselves to the stage-based relationships linking perceived visual attention, curiosity, and museum-oriented motivation in a visual-first, pre-consumption context.

The AIDA model is one of the most influential stage-based frameworks for describing how audience responses may unfold from initial attention to action [38]. Originally developed for advertising and consumer persuasion contexts, the model conceptualises audience response as a sequential transition from Attention (A), to Interest (I), to Desire (D), and then to Action (A). The AIDA model has been widely applied in marketing, tourism promotion and visual communication [39–41]. However, in non-profit museum contexts, the stage logic of AIDA is re-positioned to organise individuals’ responses to cultural stimuli, rather than to explain commercial conversion outcomes. At the same time, it does not explicitly specify the relationship between attention and motivational orientation, particularly in visually mediated, pre-consumption contexts. In such contexts, the relationship between “interest” and “desire” cannot be assumed to be direct, but may involve a distinct epistemic component. Here, “non-profit” is used to indicate that public museums primarily pursue public cultural service rather than commercial sales. In this study, non-profit is treated as a boundary condition rather than an explanatory variable. It clarifies the goal orientation of the later stages of the AIDA model: “Desire” and “Action” are interpreted in terms of cultural participation (i.e., museum visitation) rather than commercial purchase. Accordingly, in the non-profit museum context, the Desire stage of the AIDA framework is interpreted as visit motivation, reflecting an orientation toward cultural participation rather than commercial purchase.

Beyond redefining the goal orientation of the later stages, adapting the AIDA model to a visually driven, non-profit museum context also requires reconsideration of how earlier cognitive stages are conceptualised. In the context of museum-themed CCDs, two conceptual tensions emerge when applying the classic AIDA framework. The first tension concerns the interest stage, which assumes a broad cognitive engagement. The second tension concerns the Desire stage. In the original AIDA framework, desire primarily refers to commercial purchase intention, reflecting a transactional orientation toward ownership or consumption [38]. This conceptualisation does not fully apply to non-profit museum contexts, where audience responses are oriented toward experiential and meaning-oriented encounters rather than consumption [42,43]. In such contexts, visit motivation reflects a desire to encounter the source of cultural meaning referenced by CCDs, rather than to possess the product itself. This motivation can be understood as being closely linked to epistemic curiosity associated with visually mediated cultural symbols, and is oriented toward seeking an authentic and materially grounded encounter within the museum environment [43,44]. In this sense, desire is redirected from an ownership-based goal to an experience-based goal, providing a context-appropriate interpretation of the Desire component within the adapted AIDA framework.

Based on these considerations, this study conceptualises a stage-based relational framework comprising perceived visual attention (VA), curiosity (CU), visit motivation (VM), and behavioural intention (BI). This framework does not seek to replace the AIDA model, nor does it simply relabel its stages. Instead, it analytically highlights the role of information-gap-driven curiosity in linking attention with visit-oriented motivation in the specific context of image-mediated museum communication. In doing so, it clarifies a stage-based relational structure linking perceived visual attention with visit-related motivation, a pattern that remains under-specified in conventional stage-based interpretations. In such contexts, the relationship between attention and motivation is not directly observed, but is reflected in associations involving epistemic curiosity.

### 2.2. Research hypotheses

Building upon the VCVB framework conceptualised in Section 2.1 and informed by the broad stage logic of AIDA, this study outlines a set of empirically testable research hypotheses examined using PLS-SEM. In addition, the visual entry condition of the VCVB framework is articulated as a conceptual premise that clarifies the role of visual appearance at first exposure, while H2–H6 correspond to empirically testable stage-to-stage relationships.

While insights from stage-based models like AIDA have informed adjacent domains such as food-related tourism, visual communication, and museum engagement, existing insights remain fragmented across these adjacent literatures [10,45,46]. More specifically, research in food tourism and symbolic food consumption has highlighted how food products operate as cultural signifiers, through which heritage meanings, imagined authenticity, and culturally valued symbols are communicated and commodified, often through visual resemblance or symbolic association rather than direct sensory experience [47–49]. However, research on gastronomic or food-related tourism has predominantly focused on post-consumption, taste-driven experiences [25,50], while studies in visual communication and digital imagery demonstrate that visual novelty and symbolic salience can capture attention [17,26,27]. Hence, both streams pay limited attention to visually mediated, pre-consumption encounters involving hybrid stimuli that combine edible forms with museum-endorsed cultural artefacts [46,51,52]. Meanwhile, museum and heritage studies have acknowledged the importance of curiosity in learning and museum-related responses, but rarely model it explicitly as an epistemic construct linking visual perception to visit-related motivation [19,43,53,54].

Taken together, an important gap remains at the level of stage-based explanation regarding how initial visual attention to such hybrid stimuli is related to epistemic curiosity and, in turn, to behavioural intention in a pre-consumption museum context. As a result, the stage-based relationships linking visually reconstructed cultural artefacts in edible form with epistemic curiosity and museum-oriented behavioural intention remain insufficiently specified, particularly in terms of how visually induced attention is associated with subsequent motivation and behavioural responses. Specifically, H2–H4 specify the direct relationships between adjacent stages within the VCVB framework (VA → CU, CU → VM, and VM → BI), while H5–H6 assess the two indirect relationships between adjacent stages implied by this relational structure. The following subsections elaborate on these hypotheses by detailing the theoretical rationale for each proposed relationship.

#### 2.2.1. Visual appearance as the visual entry premise of the VCVB framework.

In this study, perceived visual attention is not intended to represent actual attentional allocation or cognitive resource deployment, but rather participants’ subjective appraisal of a stimulus as visually attention-worthy at first encounter. This appraisal reflects whether the stimulus is experienced as salient enough to invite further cognitive processing, rather than how attention is objectively distributed. Perceived visual attention captures the subjective experience of a stimulus’s attentional pull, distinct from objective measurement [22]. In digital environments, stimuli that are novel, realistic, or symbolically rich are perceived as more attention-grabbing [55,56]. This is amplified for visually reconstructed cultural symbols, where mimicry and realism heighten perceptual salience [27,34,57,58]. However, a critical gap persists regarding a unique hybrid stimulus: museum-themed CCDs. As edible artefacts that circulate primarily as images, their reception is fundamentally “visual-first”. Yet, prior research has rarely examined how the visual appearance of museum-themed, artefact-inspired edible products may be treated as a visually salient entry condition for the initial attention stage of audience response.

Accordingly, the present study deliberately focuses on visual-only first-exposure conditions, thereby foregrounding visually driven perceptual conditions that precede multimodal processing or image–text integration, which typically characterise later stages of museum interpretation. To clarify the analytical scope of the VCVB framework, this study treats the visual appearance of CCDs as a conceptual entry condition associated with perceived visual attention under first-exposure contexts. Accordingly, the following conceptual premise is articulated:

**Conceptual premise**: The visual appearance of museum-themed CCDs is treated as the visual entry condition associated with perceived visual attention in first-exposure contexts.

Operationalisation and Model Note: Given that the visual appearance of CCDs was intentionally standardised at the stimulus design level and not manipulated as an independent variable, the present study does not empirically examine whether variation in visual appearance leads to differences in perceived visual attention. Instead, perceived visual attention is treated as a subjectively registered entry condition under first-exposure circumstances. The analytical focus of the study is therefore placed on the stage-based relationships observed after perceived visual attention (H2–H6), rather than on how attention is generated. Accordingly, the proposed VCVB framework is a partial analytical framework that begins from perceived visual attention and does not constitute a full causal model from visual design features to behavioural outcomes.

#### 2.2.2. Visual attention and curiosity.

Perceived visual attention is conceptualised as a subjective entry point associated with viewers’ cognitive response to visually distinctive stimuli [55]. More specifically, contemporary visual cognition research shows that visual stimuli characterised by novelty, incongruence or symbolic complexity tend to be perceived as more salient and engaging, and more likely to involve deeper cognitive appraisal rather than passive noticing [19,56,59]. This attentional engagement is associated with conditions under which curiosity may emerge.

Building on this attentional foundation, curiosity is now widely conceptualised as comprising two related but distinct forms: perceptual curiosity, which is associated with visually novel or unexpected features, and epistemic curiosity, which reflects a desire to acquire knowledge or resolve informational gaps [19,60–62]. In this study, epistemic curiosity is operationalised as respondents’ information-seeking desire to identify the referenced artefact and to explore its associated cultural-historical meaning. In visually dominated media environments such as social media image streams and museum-related visual communication, perceptual curiosity is typically initiated when a stimulus is perceived by viewers as visually anomalous or attention-grabbing, after which epistemic curiosity is associated with individuals’ tendency to seek meaning, symbolism or underlying cultural information [63–65]. In digitally mediated first-exposure contexts, this pattern is often associated with the experience of an unresolved information gap, reflected in a sense of incompleteness and epistemic inquiry into the object’s identity and cultural meaning. This distinction is consistent with empirical operationalisations of curiosity in visual and digital media research, where epistemic curiosity is typically captured through information-seeking intentions rather than affective liking [66–68]. Accordingly, the CU construct operationalises epistemic curiosity (information-seeking about the referenced artefact and its cultural meaning), rather than a diffuse affective interest or hedonic liking, which aligns with its conceptual role in museum-oriented intention formation.

Furthermore, in this study, perceptual curiosity is not conceptualised as an independent latent construct but as a theoretically prior component embedded within the perceived visual attention stage. Instead, the analytical focus is placed on epistemic curiosity as the relational-level construct linking visual salience with culturally oriented motivation. This modelling choice reflects an analytical abstraction within the relational framework rather than a conceptual omission, allowing the study to isolate the information-gap–related cognitive component most proximal to visit motivation in museum contexts.

Within the context of CCDs, perceived visual attention is associated with curiosity, particularly in contexts involving visual novelty, artefact-like realism and cultural symbolism. As discussed in Section 2.1, this pattern is closely linked to the visual and symbolic incongruity of CCDs, in which an edible everyday form visually references culturally significant artefacts, creating an interpretive tension that is associated with epistemic exploration. This incongruity creates a visual–cognitive discrepancy (i.e., a mismatch between expected and perceived visual cues) that is associated with deeper exploration, as audiences may engage with questions regarding what the object represents, how it relates to cultural heritage, and why it is presented in edible form [63,64]. In museum contexts, such visually induced epistemic engagement has been shown to be closely associated with the initial phase of museum-related response and meaning-making [43,69,70].

To summarise, this relational structure can be described as an ordered set of stage-based relationships: visually distinctive stimuli may first be perceived as attention-worthy, and this perceived attentional pull may subsequently be associated with the presence of curiosity through further cognitive appraisal. Accordingly, the following hypothesis is proposed:

**H2**: Perceived visual attention is positively associated with the curiosity of potential museum visitors.

#### 2.2.3. Curiosity and visit motivation.

Curiosity is increasingly recognised in contemporary cognitive and behavioural research as being closely associated with intrinsically motivated forms of response. It can be understood as a psychological state associated with the transition from attention to an internally oriented desire to engage further [60,61,71]. Existing research shows that when individuals perceive a meaningful knowledge gap or encounter stimuli that are novel, culturally expressive or symbolically rich, curiosity often motivates efforts to reduce uncertainty through exploration or direct experience [35,72]. This pattern corresponds to a transition from epistemic wanting (“wanting to know”) to experiential orientation (“wanting to experience”), thereby suggesting a relationship between curiosity and goal-directed behavioural motivation [73,74].

Within cultural and museum contexts, curiosity has been identified as an important factor associated with visit motivation [43,69]. Research in museum learning and visitor behaviour demonstrates that epistemic curiosity is associated with individuals’ tendency to seek authentic encounters, deepen understanding and pursue experiential fulfilment, all of which constitute core components of visit motivation [43,44,75]. This pattern is particularly salient when the stimulus combines visual appeal with cultural meaning, as audiences may be **a**ssociated with a shift from passive observation to an increased interest in “seeing the real object” or “being present in the cultural setting” [44,65,69].

CCDs represent such a stimulus. Their visual novelty, artefact-like realism and symbolic references to museum collections are associated with curiosity, which in turn is associated with a stronger desire for an on-site experience. Rather than remaining at the level of mediated cultural recognition, audiences may develop an increased motivation to visit the museum, verify authenticity, or encounter the cultural artefact that the dessert visually represents. This association is consistent with the adapted AIDA framework, in which curiosity represents a cognitive component that precedes the formation of culturally grounded visit motivation.

Accordingly, the following hypothesis is proposed:

**H3**: Curiosity is positively associated with the visit motivation of potential museum visitors.

#### 2.2.4. Visit motivation and behavioural intention.

Visit motivation is conceptualised as a goal-oriented psychological state associated with individuals’ orientation towards desired cultural or experiential outcomes [76–78]. In museum visitation contexts, this motivation is associated with the expression of intention to seek authentic encounters or fulfil cultural curiosity, thereby contributing to subsequent action planning [79,80].

Behavioural intention represents the individual’s self-reported readiness to perform a future action and is widely used as an indicator of potential behaviour [79,81]. When visit motivation is strong, individuals may be more likely to express their interest in the form of concrete behavioural intentions, such as planning a visit, allocating time, or seeking additional information [82].

In the context of CCDs, this pattern is consistent with the adapted AIDA logic. The visual novelty and cultural symbolism of CCDs are associated with attention and curiosity, which in turn are associated with a culturally oriented visit motivation. When such motivation is present, individuals may form specific behavioural intentions such as planning to visit the museum, sharing the dessert imagery online, or recommending the experience to others [83]. This association reflects a shift corresponding to the Desire–Action sequence in the AIDA model, where motivational orientation is expressed in a readiness for action-oriented intention [39,84].

To sum up, visit motivation is associated with behavioural intention and reflects how motivational orientation may be expressed in planned action. Accordingly, the following hypothesis is proposed:

**H4**: Visit motivation is positively associated with behavioural intention.

#### 2.2.5. The indirect associations of curiosity and visit motivation.

Curiosity and visit motivation are proposed as key psychological constructs associated with the linkage between perceived visual attention and behavioural intention within a staged perceptual–cognitive–motivational pattern [39,84]. Building on the adapted AIDA logic, these two constructs can be described as forming a perceptual–cognitive–motivational sequence associated with visually driven cultural communication.

Perceived visual attention is associated with initial subjective cognitive response, which may in turn involve further cognitive appraisal [56]. When such attention is present, individuals may evaluate novelty, cultural meaning or symbolic features embedded in the stimulus, thereby being associated with the presence of curiosity [35,56,60]. Curiosity can be understood as a cognitive component within this sequence. It is associated with an epistemic orientation towards resolving uncertainty and acquiring additional cultural understanding, and can be understood as a cognitive component linking perceptual interest with motivation-oriented responses [19,71].

Visit motivation constitutes the next stage in this sequence. It is associated with the shift from epistemic wanting to experiential wanting and is associated with individuals’ orientation towards culturally meaningful action such as museum visitation [76,84]. In museum contexts, curiosity has been consistently found to be associated with individuals’ desire for authentic encounters, deeper interpretive engagement and greater readiness for participation [33,43]. Accordingly, visit motivation can be understood as a motivational component in which curiosity is associated with concrete behavioural intention.

Taken together, this study proposes a stage-based pattern of relationships in which perceived visual attention is associated with curiosity, which in turn is associated with visit motivation, and subsequently with behavioural intention. This relational structure clarifies how visually communicative cultural products are associated with visit-related intention through a staged process.

Based on this framework, the following hypotheses are proposed:

**H5**: Perceived visual attention is indirectly associated with visit motivation through curiosity.

**H6**: Curiosity is indirectly associated with behavioural intention through visit motivation.

## 3. Research methodology

### 3.1. Research design

This study adopts a quantitative research approach to examine how the visual presentation of museum-themed CCDs is associated with the behavioural intentions of potential visitors. It should be noted that “visual attention” in this study refers to participants’ self-reported perception of the attention-attracting quality of the CCD images, rather than objectively measured attentional allocation (e.g., eye-tracking or dwell time). Accordingly, the VA construct is operationalised as a perceptual evaluation of visual salience and attention-worthiness at first exposure, rather than as a measure of attentional intensity or duration.

In this study, the standardisation and internal validity of image-based stimuli were addressed through multiple design control measures. Image-based stimuli are widely used in tourism and visual communication research to elicit responses under controlled viewing conditions [85–87]. Building on this approach, the present study applied controlled exposure conditions to ensure that participants’ responses reflected initial subjective visual impressions rather than prior familiarity, thereby providing a basis for examining visually driven relational patterns under controlled first-exposure conditions.

To enhance the contextual relevance and real-world applicability of the survey questionnaire, image-based stimuli were incorporated to simulate the scenario in which the public first encounters CCD images on social media platforms. Accordingly, four widely circulated and representative CCDs were selected from social media as visual cues, each corresponding to a typical category of museum artefacts: pottery, porcelain, metal ornaments, and bronze ware. These four categories represent core artefact exhibition types commonly found in museums and offer both representativeness and visual diversity. This allows for a more comprehensive representation of visually driven perceptual responses. The CCDs featured in the survey were officially launched by institutions such as the National Museum of China and the Henan Museum. The dessert designs or presentations faithfully replicate key features of the original artefacts, combining strong cultural symbolism with high recognisability in visual communication.

Before answering the questionnaire, all respondents were required first to view the images to simulate the initial visual exposure they would encounter in real social media environments. Additionally, to eliminate the influence of prior exposure on visual judgement and behavioural intention, only respondents encountering these culturally creative dessert images for the first time were permitted to proceed. This approach ensured that responses were based on initial visual impressions, thereby improving the alignment between the data and the stage-based relationships examined in this study. Eligibility was determined through screening items embedded in the questionnaire system. Those not meeting the criteria were automatically excluded from completing the survey. This design focuses on the early-stage relationships proposed in the VCVB framework, beginning from perceived visual attention under first-exposure conditions.

**Fig 2:**
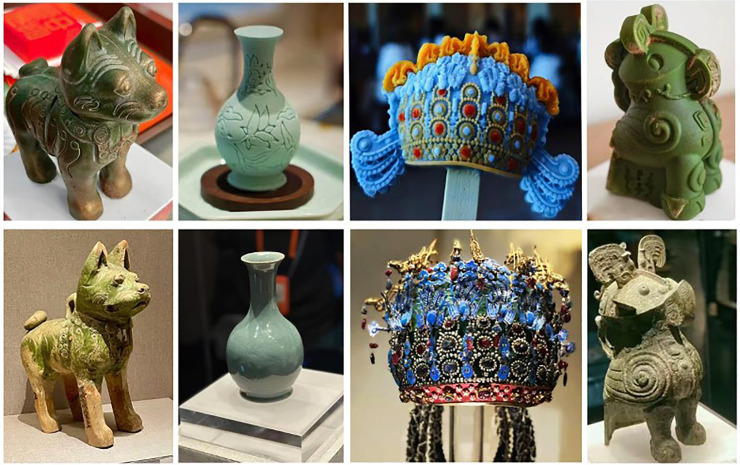
Visual representations of four CCD dessert images and their corresponding artefacts (Photographs by the author).

The original images of the artefacts were not shown to participants during the actual survey. This was done to allow respondents’ reactions to focus primarily on the visual appearance of the CCDs, without being influenced by perceived resemblance or other external contextual information. The CCD images were presented without textual identification of the artefacts to capture participants’ inferences based on visual cues. Fig 2 presents the four CCD dessert images used in the questionnaire. The CCD dessert images were selected to clearly present their form, colour, and artefact-like features, while avoiding the inclusion of price information, textual descriptions, or other background cues that might distract from visual evaluation. The corresponding original artefact images are included solely for academic illustration and reader reference. These CCDs were identified based on their wide circulation on social media platforms, and the images used in the survey were photographed by the author on-site to ensure consistency and copyright clarity.

### 3.2. Research instrument & data collection

This study employed a structured questionnaire to measure four latent variables, including perceived visual attention, curiosity, visit motivation, and behavioural intention. The questionnaire consisted of two parts. The first part focused on structural measurement and included four latent constructs: perceived visual attention (VA), CU, VM, and BI. Each construct was measured using three items, all evaluated on a five-point Likert scale (1 = strongly disagree, 5 = strongly agree). The items were adapted from several established studies, including Weng et al. on advertising responses [45], Wei et al. on sensory marketing and visitor behavioural intention [41], Byun and Jang on the influence of destination advertising [88], Hassan et al. on the application of the AIDA model in social media strategies [89], and Ponte et al. on the effects of trust and perceived value on tourism behaviour [90]. The specific items are listed in Table 1. Consistent with prior survey-based studies in visual communication, perceived visual attention was operationalised as respondents’ perceived attention attraction and visual salience of the CCD images, captured through self-report items. VA includes both attention attraction and artefact-related visual salience/association as part of perceived visual attention in this study, consistent with the visually reconstructed cultural characteristics of CCDs. Although one item refers to visual attractiveness, the VA construct as a whole is intended to capture perceived attention-worthiness and culturally salient visual appraisal rather than aesthetic liking per se.

**Table 1:** Measurement items for latent variables.

Latent Variable	Measurement Item
**Perceived Visual Attention (VA)**	The visual appearance of the desserts is highly attractive.
	The design of the desserts is visually distinctive and creative.
	The designs of these desserts remind me of traditional artefacts or historical relics.
**Curiosity (CU)**	I am curious about which artefact or relic these desserts are modelled after.
	I want to further explore the culture and history behind the selected artefacts.
	The visual design of these desserts has increased my desire to learn more about the associated museum.
**Visit Motivation (VM)**	I hope to see the actual dessert creations in person.
	I hope to visit and see the original artefacts that inspired these desserts.
	The visual presentation of these desserts has strengthened my motivation to visit the associated museum.
**Behavioural Intention (BI)**	If time and financial conditions allow, I intend to visit the museum in the near future.
	I am willing to share my visit experiences related to the museum and the desserts on social media.
	If I have a satisfying experience at the museum, I am willing to recommend it to friends and family

The second part of the questionnaire covered demographic information and relevant background variables, including gender, age, educational background, museum visitation frequency, and other relevant background information. The questionnaire was initially drafted in English. To ensure semantic accuracy and respondent accessibility, the final version was administered in Simplified Chinese.

This study targeted individuals aged 18 and above with basic Chinese reading proficiency as respondents. Before the official release of the survey questionnaire, a pilot test was conducted to assess the clarity of items and their logical coherence [91,92]. The questionnaire was distributed online using the same method as the formal survey. The recruitment period for the pilot study lasted from 02/04/2025 to 04/04/2025. A total of 40 valid responses were collected and used to conduct reliability analysis of the measurement scales. The Cronbach’s alpha coefficients for all latent variables exceeded the recommended threshold of 0.70, indicating good internal consistency and reliability of the measurement scales [93]. As a result, all items were retained for the actual survey. These procedures supported the internal consistency and overall quality of the questionnaire prior to formal data collection.

The formal data collection began on April 25, 2025, and ended on May 1, 2025, lasting for one week. Following commonly adopted guidelines for PLS-SEM, a sample size of 100–200 responses is generally considered sufficient for stable model estimation and evaluation [94–96]. Based on these guidelines, the study set a target sample size of 200. The questionnaire was distributed using snowball sampling via the researcher’s personal networks, WeChat groups, and other social media platforms to expand the reach of potential participants.

This sampling strategy resulted in a sample composed primarily of individuals active in digital and social media environments, which aligns with the study’s analytical focus on visually mediated encounters with CCD imagery in online contexts. Accordingly, the findings are confined to the stage-based relationships specified by the VCVB framework under first-exposure conditions.

To address potential common-method variance, several procedural remedies were applied, including ensuring respondent anonymity. Items were kept concise and neutral, and respondents were instructed to answer based on their immediate impressions after viewing the images. In addition, demographic items were placed in a separate section.

A total of 274 responses were collected during the data collection period. Responses that were incomplete or failed to meet the predefined eligibility criteria, as assessed through screening items embedded in the questionnaire system (e.g., prior exposure to the CCD images), were automatically excluded. As a result, 205 valid responses were retained for analysis, yielding a valid response rate of 74.8%.

### 3.3. Ethics statement

The study was conducted in accordance with the Declaration of Helsinki and received ethical approval from the Ethics Committee of Anyang Institute of Technology, Henan Province, China (Approval Code: AG/ECS2503001), prior to data collection. Data were collected using an online questionnaire hosted on a secure survey platform. Participants were recruited using snowball sampling via the researcher’s personal networks, WeChat groups, and other social media platforms, which were used solely to disseminate the survey link. As the study was non-interventional, participants were informed that their participation was voluntary and that their identities would remain anonymous. All data were anonymised prior to analysis and dissemination and were used for academic research purposes. An informed consent statement was embedded at the beginning of the online questionnaire. Participants were required to read the statement, which outlined the purpose of the study and the voluntary nature of participation. Consent was indicated by clicking the “Agree” button, after which access to the questionnaire was granted. To ensure compliance with age-related ethical standards, the survey system was configured to allow only participants aged 18 years or above to proceed. No personally identifiable information was collected or disclosed at any stage of the study.

### 3.4. Data analysis

In this study, SPSS 26 was initially used to conduct descriptive statistical analysis of the respondents’ demographic characteristics and relevant background information. Descriptive statistics were used solely to summarise respondents’ background characteristics, while hypothesis testing and the validation of the proposed research model were conducted through PLS-SEM analysis. For multivariate statistical modelling and analysis, SmartPLS 4.0 was employed to construct the PLS-SEM, which was used to assess the relationships between latent variables and to evaluate the measurement and structural models. In addition, the reliability and validity of the measurement model were evaluated through standard assessments, including reliability, convergent validity, and discriminant validity.

For the evaluation of the measurement model’s validity, indicator items with standardised factor loadings greater than 0.7 were prioritised for retention [97]. Items with loadings between 0.4 and 0.7 were retained only if their inclusion did not significantly reduce the composite reliability (CR) or the average variance extracted (AVE). An AVE threshold of 0.5 was adopted to determine convergent validity. Composite reliability (CR) and Cronbach’s alpha were applied for reliability assessment, with the recommended cutoff value for both being 0.7 [97]. Discriminant validity was assessed using the Fornell-Larcker criterion, which requires that the square root of each construct’s AVE exceed its correlations with other constructs. Additionally, the Heterotrait-Monotrait Ratio (HTMT) was applied as a supplementary measure of discriminant validity, with values below the threshold of 0.85 considered acceptable [98].

In the structural model testing phase, the bootstrapping method was employed to analyse the significance of path coefficients, with the number of sub-samples set to 5,000. The analysis reported the path coefficients (β), t-values, p-values, and 95% bias-corrected confidence intervals (CI). The model’s explanatory capacity was evaluated using R² values, while effect sizes were supplemented with f² values. All path analyses were conducted using two-tailed tests, with a significance level set at 0.05 [97]. Common-method variance was assessed using the full collinearity VIF approach in SmartPLS [97,99]. All VIF values were below the recommended thresholds, suggesting that common-method bias was not a major concern in this study.

### 3.5. Profile of respondents

Table 2 provides a descriptive summary of the demographic characteristics of the respondents, including gender, age, educational background, museum visitation frequency, and prior exposure to museum cultural and creative products. This information serves to contextualise the sample rather than to test the research hypotheses. Museum visitation frequency categories (Rarely, Sometimes, Frequently) were operationalised based on self-reported visit frequency.

**Table 2:** Respondents’ profile.

Variables		Frequency (n = 205)	Percentage (%)
Gender	Male	109	53.2
	Female	96	46.8
Age Group	18-29 years old	49	23.9
	30-39 years old	75	36.6
	40-49 years old	53	25.9
	50 years old and above	28	13.7
Education	High school or below	19	9.3
	Diploma/ Associate degree	124	60.5
	Bachelor’s degree	42	20.5
	Master’s degree or above	20	9.8
Frequency of museum visits	Never	12	5.9
	Rarely (Less than once per year)	105	51.2
	Sometimes (1–2 times per year)	62	30.2
	Frequently (At least 3 times per year)	26	12.7
Experience with museum cultural products	Never experienced	17	8.3
	Seen but not purchased	74	36.1
	Purchased or owned	114	55.6

A total of 205 valid responses were collected in this study. Among the respondents, 53.2% were male and 46.8% were female. In terms of age distribution, the majority of respondents were aged 30–39 (36.6%), followed by those aged 40–49 (25.9%) and 18–29 (23.9%), with proportions relatively close. Respondents aged 50 and above accounted for the smallest group at 13.7%. Regarding educational background, over 60% (60.5%) of the respondents held a diploma or associate degree, followed by those with a bachelor’s degree (20.5%) and those with a master’s degree or above (9.8%).

In terms of museum visit frequency, 51.2% of respondents indicated that they visited museums less than once per year (Rarely), 30.2% reported visiting 1–2 times per year (Sometimes), and 12.7% visited at least three times per year (Frequently). Only 5.9% reported that they had never visited a museum. Overall, the sample includes respondents with varying levels of museum visitation experience.

In terms of exposure to cultural and creative products from museums, 55.6% of respondents stated that they had purchased or owned such products, while 36.1% had seen them but did not purchase. Only 8.3% had never encountered these products. These results suggest that most respondents have had some level of exposure to museum-related cultural and creative products.

## 4. Findings and results

### 4.1. Measurement model

As shown in Table 3, all latent variables demonstrated strong reliability, with Cronbach’s alpha (α) values exceeding 0.7 (ranging from 0.743 to 0.821), indicating good internal consistency for each construct. The composite reliability (CR) values were all above 0.8 (ranging from 0.853 to 0.893), surpassing the conventional threshold of 0.7, further confirming the measurement stability of each latent construct. For clarity, although the construct is labelled as “Visual Attention (VA)” in the measurement and structural models, it consistently refers to participants’ perceived visual attention as defined in Section 3, rather than objectively measured attentional allocation.

**Table 3:** The measurement model.

Latent Variable	Item	Factor Loadings	Cronbach’s Alpha (α)	Composite Reliability (CR)	AVE
VA	VA1	0.805	0.743	0.853	0.660
	VA2	0.770			
	VA3	0.860			
CU	CU1	0.890	0.821	0.893	0.736
	CU2	0.845			
	CU3	0.839			
VM	VM1	0.838	0.753	0.858	0.668
	VM2	0.807			
	VM3	0.807			
BI	BI1	0.792	0.751	0.857	0.667
	BI2	0.824			
	BI3	0.833			

Regarding convergent validity, all standardised factor loadings were above 0.7 (ranging from 0.770 to 0.890), and the AVE values for all constructs exceeded the threshold of 0.5 (ranging from 0.660 to 0.736). These results indicate that all measurement items adequately reflect their corresponding latent constructs, supporting the adequacy of convergent validity.

As shown in Table 4, the square root of each construct’s AVE was greater than its correlations with other constructs, satisfying the Fornell-Larcker criterion and indicating adequate discriminant validity. To further verify this, the HTMT ratio of correlations was also examined. As shown in Table 5, all HTMT values were below the recommended threshold of 0.85 [98], thereby further supporting the discriminant validity of the constructs.

**Table 4:** Discriminant validity.

	VA	CU	VM	BI
VA	**0.812**	0.435	0.123	0.030
CU	0.435	**0.858**	0.404	0.311
VM	0.123	0.404	**0.817**	0.561
BI	0.030	0.311	0.561	**0.817**

**Note:** Diagonal elements represent the square roots of the average variance extracted (AVE). Off-diagonal elements represent inter-construct correlations.

**Table 5:** HTMT of correlations.

	VA	CU	VM	BI
VA	–	0.550	0.216	0.071
CU	0.550	–	0.488	0.384
VM	0.216	0.488	–	0.749
BI	0.071	0.384	0.749	–

**Note**: All HTMT values were below the recommended threshold of 0.85, supporting the discriminant validity of the constructs.

It is worth noting that in the subsequent structural model analysis (Section 4.2), a supplementary direct relationship between VA and VM was included to assess the robustness of the proposed relational structure. Since this adjustment did not involve any changes to the latent constructs or their measurement items, the previously reported reliability and validity assessment remain applicable according to the recommendations by Hair [97]. Therefore, re-evaluation of the measurement model was not required.

### 4.2. Structural model and hypothesis testing

Before hypothesis testing, collinearity among predictor constructs in the structural model was assessed using inner Variance Inflation Factor (VIF) values. All inner VIFs were well below the recommended threshold (VIF < 5), indicating that multicollinearity was not a concern [97].

**Fig 3:**
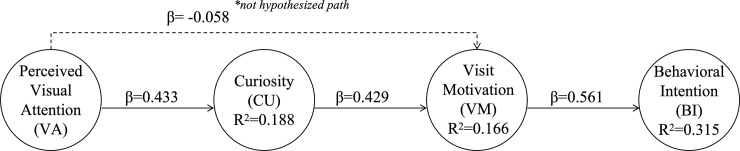
Structural model and relationship estimates within the VCVB framework. **Note:** The direct relationship between Visual Attention (VA) and Visit Motivation (VM) was not hypothesised a priori and was included as a supplementary diagnostic check to assess the robustness of the proposed relational structure. Accordingly, this relationship is reported for completeness but is not interpreted as a formal hypothesis test.

As shown in Table 6 and Fig 3, all hypothesised direct relationships (H2-H4) were statistically significant. Perceived visual attention was positively associated with curiosity (VA → CU), curiosity was positively associated with visit motivation (CU → VM), and visit motivation was positively associated with behavioural intention (VM → BI).

**Table 6:** Structural model results and hypothesis assessment.

Relationship	β	T	f^2^	95% CI	Hypothesis	Supported
Direct relationships						
VA → CU	0.433	6.108	0.231	[0.281,0.553]	H2	Yes
CU → VM	0.429	6.381	0.180	[0.279,0.548]	H3	Yes
VM → BI	0.561	11.366	0.460	[0.451,0.650]	H4	Yes
*VA → VM	−0.058	0.826	0.003	[-0.192,0.086]	N/A	Not hypothesised
Indirect relationships						
VA → CU → VM	0.186	4.922	–	[0.118,0.265]	H5	Yes
CU → VM → BI	0.241	5.502	–	[0.152,0.322]	H6	Yes

**Note:** Hypothesised relationships (H2–H6) were tested using 5,000 bootstrap resamples with 95% bias-corrected confidence intervals. The VA → VM relationship was included as a supplementary diagnostic check to assess the robustness of the proposed relational structure and was not hypothesised a priori. Accordingly, this relationship is reported for completeness of the structural assessment but is not interpreted as a formal hypothesis test.

According to Cohen’s guidelines, an f² value of 0.02 indicates a small effect, 0.15 a medium effect, and 0.35 a significant effect [100]. In this study, the f² effect size analysis indicated medium effects for VA → CU and CU → VM, and a relatively larger effect size for VM → BI (see Table 6).

Bootstrapping results indicated significant indirect relationships for VA → CU → VM and CU → VM → BI, supporting H5 and H6. Taken together, these results are consistent with a staged curiosity–motivation relational pattern linking perceived visual attention with behavioural intention, as reflected in the statistically significant relationships between VA and CU, CU and VM, and VM and BI. The R² values were 18.8% for CU, 16.6% for VM, and 31.5% for BI, indicating a moderate level of variance explained by the model. Overall, the results indicate that perceived visual attention is associated with visit motivation indirectly through curiosity, while the direct association between visual attention and visit motivation is not statistically significant. Behavioural intention was most strongly associated with visit motivation, with perceived visual attention linked to behavioural intention through the sequential pathway of curiosity and visit motivation. This pattern is consistent with the study’s core theoretical argument that, in first-exposure image-mediated museum contexts, the relationship between attention and motivation is not direct but operates through epistemic curiosity.

### 4.3. Comparative modelling analysis

To assess whether the proposed VCVB framework provides additional explanatory value beyond simplified structures, a comparative modelling analysis was conducted. In addition to the proposed full model (VA → CU → VM → BI), two alternative models were estimated: a direct effect model (VA → BI) and a reduced mediation model (VA → VM → BI).

The results (see Table 7) indicate systematic differences in model specification and explanatory patterns across the three models. The direct effect model (Model 1) shows that perceived visual attention is not significantly associated with behavioural intention (β = 0.076, t = 0.464, p = 0.643), with minimal explanatory power (R² = 0.006). This suggests that visual attention alone is insufficient to explain behavioural intention in this context.

**Table 7:** Comparative model specifications and summary results.

Model	Structure	Path coefficients (β, t, p)	R² (BI)	Interpretation
Model 1	VA → BI	VA → BI: β = 0.076, t = 0.464, p = 0.643	0.006	Non-significant direct effect model
Model 2	VA → VM → BI	VA → VM: β = 0.177, t = 2.662, p = 0.008 VM → BI: β = 0.566, t = 11.596, p < 0.001	0.321	Motivation-mediated model
Model 3	VA → CU → VM → BI	VA → CU: β = 0.433, t = 6.108, p < 0.001 CU → VM: β = 0.429, t = 6.381, p < 0.001 VM → BI: β = 0.561, t = 11.366, p < 0.001	0.315	Full VCVB model (proposed sequential mediation model)

Introducing visit motivation as an intermediate construct (Model 2) improves the explanatory capacity of the model. Perceived visual attention is significantly associated with visit motivation (β = 0.177, t = 2.662, p = 0.008), which in turn is strongly associated with behavioural intention (β = 0.566, t = 11.596, p < 0.001). The explanatory power for behavioural intention increases substantially to R² = 0.321. However, the relatively low R² for visit motivation indicates that visual attention alone provides limited explanation of motivational orientation.

The full VCVB model (Model 3) further extends this structure by incorporating epistemic curiosity as an intermediate cognitive component. All structural relationships (VA → CU, CU → VM, VM → BI) are statistically significant (p < 0.001), and the model explains 31.5% of the variance in behavioural intention (R² = 0.315). Although this explanatory power is comparable to that of Model 2, the inclusion of curiosity provides a more theoretically specified account of how visual attention is associated with behavioural intention through a staged perceptual–cognitive–motivational process.

This pattern further suggests that the commonly assumed direct linkage between visual attention and motivational orientation does not hold in this context. Instead, the proposed VCVB framework offers a more contextually grounded and structurally articulated explanation by incorporating epistemic curiosity as a key intermediate component.

Furthermore, consistent with the structural model results reported in Table 6, the relationship between perceived visual attention and visit motivation is not directly observed in the full model, but is reflected through curiosity. This reinforces the interpretation that epistemic curiosity functions as a key intermediate component linking attention with motivational orientation in visually mediated museum contexts.

## 5. Discussion

The present study adopts a structure-oriented perspective to examine how visual attention is linked to visit motivation under first-exposure conditions. Accordingly, the observed R² values should be interpreted as indicating partial explanatory power within a broader motivational ecology of museum visitation, rather than as indicators of model weakness. A substantial proportion of variance in curiosity, visit motivation, and behavioural intention is likely attributable to broader background factors, such as prior museum involvement, cultural capital, or habitual social media engagement, which shape individuals’ general engagement orientations but are not the focus of the perceptual–motivational sequence examined in this study. The presence of such factors does not necessarily undermine the proposed stage-based ordering of associations (VA → CU → VM → BI), but instead highlights the conditional and non-deterministic nature of visually driven communication processes.

This study provides empirical evidence supporting the VCVB framework as a structure-oriented account of visually driven museum communication. The findings identify an ordered perceptual–motivational pattern (VA → CU → VM → BI) and, more importantly, indicate that the commonly assumed direct linkage between visual attention and visit motivation does not hold under first-exposure, image-mediated conditions. Perceived visual attention is positioned at the early perceptual stage, where it is associated with curiosity, which is subsequently linked to visit motivation as the most immediate stage associated with behavioural intention. Behavioural intention is therefore associated with perceived visual attention through an indirect pathway via curiosity and visit motivation.

Bootstrapped mediation analysis further indicates two significant indirect relationships: perceived visual attention (VA) → curiosity (CU) → visit motivation (VM) and curiosity (CU) → visit motivation (VM) → behavioural intention (BI). These findings clarify the relational pattern through which visually communicative CCD imagery is associated with behavioural intention, without assuming a direct progression from attention to action. This pattern indicates that the transition from attention to motivation is not direct, but is contingent upon an intermediate epistemic process, rather than reflecting a straightforward stage progression. It is important to note that perceived visual attention refers to respondents’ subjective appraisal of visual salience, rather than objectively measured attentional allocation, and should therefore be interpreted as a perceptual entry condition within the proposed relational structure.

The comparative modelling results further strengthen this interpretation. The findings indicate that behavioural intention is neither directly associated with perceived visual attention nor sufficiently explained by a simplified motivation-mediated structure alone. While the reduced model (VA → VM → BI) shows comparable explanatory power (R² = 0.321, compared to 0.315 in the full model), it does not provide a theoretically differentiated account of how motivational orientation emerges from visual attention. By contrast, the inclusion of epistemic curiosity in the full VCVB framework specifies an intermediate cognitive process through which visual attention is associated with visit motivation and subsequent behavioural intention. This suggests that epistemic curiosity functions as a key component in transforming perceptual responses into motivational orientation under first-exposure, visually mediated conditions.

Furthermore, curiosity is positioned as a key cognitive component within the proposed relational structure, connecting perceived visual attention with visit motivation and, subsequently, behavioural intention. Rather than acting merely as an intermediate variable, curiosity represents a distinct epistemic component that conditions how perceptual responses are associated with culturally oriented motivation. In the context of CCDs, this pattern reflects how visually mediated cultural representations may be associated with visit-related intention through non-verbal and symbolic communication. The findings highlight the role of museum-themed CCDs as visually communicative cultural products that are associated with visit-related intention through their association with epistemic curiosity, thereby extending the communicative function of museums beyond physical exhibition spaces into digitally mediated environments.

### 5.1. Implications for theory

This study contributes to theory by analytically specifying and providing empirical evidence consistent with a relational structure that is not explicitly articulated in stage-based models such as AIDA. While AIDA assumes a sequential progression from attention to action, it does not specify how attention is associated with motivational orientation in such contexts. The present findings indicate that, in visual-first, pre-consumption contexts, this association is not direct. Instead, perceived visual attention shows no significant direct association with visit motivation and is linked to behavioural intention only indirectly through epistemic curiosity. This indirect-only mediation pattern suggests that the association between attention and motivational orientation is contingent upon an intermediate epistemic component, providing empirical clarification of a relational mechanism that remains under-specified in AIDA. This finding further demonstrates that the commonly assumed direct attention–motivation linkage is not empirically supported in visual-first, pre-consumption contexts.

This absence of a direct association provides a more precise specification of conventional AIDA-based interpretations, where a progressive transition from attention to interest and subsequently to desire is typically assumed without explicitly detailing the underlying relational mechanism. By contrast, the present findings indicate that attention does not directly shape motivational orientation, but operates through an epistemic component. In this sense, the contribution lies not in extending or relabelling AIDA, but in demonstrating that the assumed attention–motivation linkage may not operate directly under visual-first conditions, thereby indicating the need for an alternative relational specification. By isolating epistemic curiosity, the study shows that the “interest” stage in AIDA involves a distinct epistemic component through which attention is associated with visit-related intention.

The comparative modelling analysis further strengthens this theoretical claim by demonstrating that simplified structural specifications fail to provide a theoretically differentiated account of behavioural intention. The non-significant direct effect observed in the direct model (VA → BI), together with the limited structural explanation of the reduced mediation model (VA → VM → BI), indicates that neither attention alone nor a simplified motivation-mediated structure sufficiently captures the intermediate cognitive process through which intention is formed. By contrast, the full VCVB model specifies a distinct relational structure in which epistemic curiosity functions as a necessary intermediate component linking visual perception with motivational orientation. This indicates that the relationship between attention and motivation is not only indirect, but structurally dependent on an epistemic process that cannot be reduced to simplified or direct specifications. This suggests that, in visual-first contexts, attention is associated with motivational orientation through epistemic curiosity within the present relational structure.

The study further clarifies the theoretical role of curiosity as a distinct epistemic component rather than a generic affective stage. By conceptualising curiosity as an information-gap-driven cognitive state that is associated with individuals’ tendency to seek meaning, knowledge, and verification [19,54,71], the analysis provides further specification of the association between visual salience and motivational orientation. This interpretation is consistent with prior research suggesting that curiosity operates as an exploratory cognitive component grounded in information-gap dynamics [35,37]. In this framework, epistemic curiosity is positioned as a necessary cognitive condition rather than a by-product of visual salience, corresponding to a state of informational insufficiency associated with active information seeking [36].

Finally, the study provides a context-bounded specification for visually mediated museum communication. The VCVB framework provides an analytical articulation of how stage-based intention formation may be structured in visual-first, pre-consumption contexts involving hybrid cultural stimuli such as CCDs. The study clarifies how visually encountered stimuli are associated with museum-oriented motivation prior to visitation, as discussed in museum communication research [9,101]. These contributions position visually driven communication as a conditional sequence in which attention functions as an entry point rather than a sufficient condition for action. In this sense, epistemic curiosity operates as a key cognitive component associated with the relationship between visual perception and culturally oriented motivation.

From the perspective of food tourism and heritage consumption, the study further contributes by examining how visually mediated, institutionally endorsed edible artefacts are associated with visit-oriented motivation under first-exposure conditions. The findings also suggest that visual appeal alone does not necessarily translate into deeper interpretive understanding, highlighting the importance of aligning sensory design with epistemic and interpretive processes.

Overall, by clarifying the role of epistemic curiosity as a necessary intermediate condition, this study provides a structure-oriented account of how image-based cultural products are associated with museum-related behavioural intention. The findings demonstrate that visual attention alone is insufficient to generate visit-related intention, and that intention formation in visual-first contexts is structurally dependent on an epistemic process linking perception to motivation.

### 5.2. Implications for practice

Building on the proposed VCVB framework and the present findings, practical implications for museum design are discussed according to the stage-based structure of visual attention (VA), epistemic curiosity (CU), and visit motivation (VM) under first-exposure conditions.

At the entry stage, visual presentation is associated with perceived visual attention. In visually mediated environments such as social media, design interventions at this stage are more closely associated with attracting attention than with directly motivating visitation. Accordingly, the findings suggest that visually salient CCDs, particularly those emphasising strong cultural symbolism and recognisable artefact references, may function as effective visual entry points within image-driven communication contexts.

At the curiosity stage, design decisions may shape whether initial attention is further associated with deeper epistemic processing or remains at the level of perceptual novelty. The findings suggest that curiosity is associated not simply with visual attractiveness, but with the presence of an information gap. Accordingly, CCDs that incorporate recognisable yet incomplete artefact cues, balancing visual fidelity with interpretive ambiguity, may be more likely to be associated with exploratory responses. This allows viewers to identify cultural references while maintaining uncertainty about their meaning, thereby encouraging further exploration.

At the visit motivation stage, the focus shifts to supporting the consolidation of curiosity into action-oriented readiness. Visit motivation functions as the proximal stage associated with behavioural intention. The findings suggest that aligning follow-up communication and interpretive support with emerging curiosity may be associated with stronger visit-related intention. For example, linking CCD imagery to accessible narratives about artefacts, or integrating CCD-related experiences into guided activities, workshops, or digital content, may be associated with the continuation of curiosity and the formation of visit-related intention. Across these stages, the key implication is that visually engaging CCDs are most effective when embedded within a broader interpretive and participatory context. Rather than functioning as stand-alone attractions, CCDs may act as entry points that support a progressive sequence from visual attention to curiosity and, ultimately, to visit-related intention.

From a broader perspective, these findings suggest that visually mediated cultural products can support museum communication by lowering the threshold for initial attention and curiosity formation, particularly in digital environments. However, their effectiveness depends on how visual design, interpretive cues, and participatory formats are aligned with the attention–curiosity–motivation pattern identified in this study. Within this scope, the present findings provide a structure-oriented perspective rather than prescriptive design solutions, and should therefore be interpreted as indicative of relational patterns rather than specific optimisation strategies.

### 5.3. Limitations and future research directions

While this study develops and provides empirical evidence consistent with a visually driven communication relational structure for museum-themed CCDs, several constraints should be understood as boundary conditions of the present study rather than limitations that undermine its analytical validity. The study is intentionally situated within first-exposure, image-mediated encounters involving artefact-referential CCDs, based on cross-sectional self-report data. This design allows the analysis to isolate visually mediated perceptual–cognitive–motivational associations under controlled conditions. Accordingly, claims regarding museum-oriented intention formation should be interpreted at the level of intention-based relational structure rather than as generalisable behavioural outcomes.

First, this study captured participants’ perceptions and attitudes at a single point in time, making it difficult to trace the dynamic evolution of behavioural intention. Future research could adopt longitudinal or experimental tracking designs to observe changes in behavioural intention following initial visual exposure, and their relationship to actual museum visit behaviour, thereby enhancing the temporal validity and empirical applicability of the VCVB framework.

Second, the present study is situated within a mainland Chinese context and focuses on first-exposure encounters with artefact-referential CCD imagery. These conditions define the contextual scope within which the proposed relational structure is examined and should not be interpreted as claims of universal applicability. In other cultural settings, while the information-gap–driven curiosity pattern may remain relevant, its expression may vary depending on cultural familiarity, symbolic interpretation, and institutional context. Similarly, for audiences with prior exposure or repeated encounters, curiosity may attenuate, and alternative relational dynamics (e.g., familiarity, affective attachment, or experiential expectation) may become more salient. In addition, visually communicative museum products that do not directly reference identifiable artefacts, or other forms of museum communication beyond CCDs, may involve different perceptual–motivational pathways. Future research is therefore encouraged to examine how the proposed relational structure varies across cultural contexts, exposure histories, stimulus types, and communication formats.

Third, the sample was recruited using snowball sampling via social media and personal networks, which may introduce self-selection bias and limit the generalisability of the findings. Accordingly, the sample should be interpreted as indicative rather than fully representative, which is consistent with the relational structure-oriented focus of this study. Future research could employ probability-based sampling designs to further examine the robustness of the proposed relational structure across different populations.

Fourth, this study focused on sensory-driven relational structures without accounting for audience characteristics such as age, gender, or education. Future research could incorporate audience attributes to strengthen the explanatory power and generalisability of the VCVB framework. In particular, future studies could adopt segmentation-based analyses to examine how different audience groups engage with the VCVB framework in distinct ways, thereby enabling more targeted and differentiated museum communication strategies.

Finally, this study examined behavioural intention rather than actual behaviour. While behavioural intention is commonly examined as an intention-level outcome in tourism research, future research should empirically examine the intention–behaviour conversion under visually driven cultural communication conditions, in order to assess the durability and real-world implications of motivation associated with CCD exposure. In addition, future research may explicitly model perceptual and epistemic curiosity as separate latent constructs to further examine their dynamic interaction in visually mediated museum communication.

In addition, perceived visual attention in this study was operationalised through self-reported perceptions of attention attraction rather than objective attentional measures such as eye-tracking or dwell-time recording. While this approach is consistent with prior survey-based research on visual communication and audience perception, it does not capture actual attentional allocation at the behavioural or physiological level. Future studies could integrate objective attention-tracking methods to further examine and extend the VCVB framework.

At a broader level, while this study highlights the potential role of visually driven communication processes, future research should further explore how museums can balance visual and experiential appeal with relational structures that foster meaning internalisation and critical cultural awareness. This includes examining how interpretive narratives, educational mediation, or participatory formats can be integrated into visually engaging products such as CCDs, in order to reduce the likelihood of surface-level responses and support deeper cultural understanding. Future research may extend the VCVB relational structure to examine how participation-oriented behavioural intention associated with visually driven cultural communication relates to broader participation-related outcomes across diverse cultural contexts.
